# Indications and Contraindications for Liver Transplantation

**DOI:** 10.4061/2011/121862

**Published:** 2011-10-05

**Authors:** Vibha Varma, Naimish Mehta, Vinay Kumaran, Samiran Nundy

**Affiliations:** Department of Surgical Gastroenterology and Liver Transplantation, Sir Ganga Ram Hospital Room No. 2221, SSR Block, Rajinder Nagar, New Delhi 110060, India

## Abstract

Patients with chronic liver disease and certain patients with acute liver failure require liver transplantation as a life-saving measure. Liver transplantation has undergone major improvements, with better selection of candidates for transplantation and allocation of scarce deceased donor organs (according to more objective criteria). Living donor liver transplantation came into existence to overcome the shortage of donor organs especially in countries where there was virtually no deceased donor programme. Advances in the technical aspects of the procedure, the intraoperative and postoperative care of both recipients and donors, coupled with the introduction of better immunosuppression protocols, have led to graft and patient survivals of over 90% in most high volume centres. Controversial areas like transplantation in alcoholic liver disease without abstinence, acute alcoholic hepatitis, and retransplantation for recurrent hepatitis C virus infection require continuing discussion.

## 1. Introduction

Liver transplantation is a life-saving procedure for patients with chronic end stage liver disease and selected patients with acute liver failure (ALF) [1–3]. Over the years, the technique of the operation has undergone major changes. Together with this, there has been an improvement in the understanding of pre- and posttransplantation physiology and the introduction of newer and more effective immunosuppressive drugs and strategies for preventing posttransplantation infections so that, in the United States, the one year patient survival has now reached 87.6% and graft survival 82.4% [4]. 

Liver grafts for transplantation can be obtained either from deceased donors (DDs) or living donors (LDs). Living donor liver transplantation (LDLT) was introduced because of the increasing demand for donor organs and the widening gap between the resource (deceased donor) and demand (recipient). It is very important to prioritize the patients for organ allocation in a deceased donor liver transplantation (DDLT) programme. This is, however, different in a programme which is based mainly on LDLT where the prospective donor is usually a close relation. However, in both the situations, a measure such as a scoring system is important in prognosticating the outcome following transplantation. There has to be a balance between the patient's medical reserves to withstand a major operation like liver transplantation and its probable outcome.

For DDLT organ, allocation was initially based on the location of the patient (at home, in hospital or in an intensive care unit) and the time on the waiting list (United Network for Organ Sharing-UNOS status). However, with the use of more objective mathematical models, based on certain selected risk factors such as the model for end-stage liver disease (MELD) score, the system of allocation has probably improved. The MELD and PELD (for paediatric recipients) scores are systems for assessing a patient's need for transplantation or for the likelihood of requiring transplantation in the future [5–7].

The older Child-Turcotte-Pugh (CTP) classification system and its variations have also been used to stratify patients with chronic liver disease to predict the mortality and morbidity. However, because it relies on many subjective criteria, its use has been superseded by the MELD and PELD scores. These are mathematical regression models which objectively assess the need for liver transplantation and more accurately predict the short-term mortality while on the transplantation waiting list. Their purpose is to help physicians select those patients who might benefit most from the transplantation. The MELD score is calculated using the patient's international normalized ratio (INR), bilirubin, and creatinine according to the formula given below [8, 9]. 

MELD score = 10{0.957 log(serum creatinine) + 0.378 log(total bilirubin) + 1.12 log(INR) + 0.643}

If the MELD score is ≥30 the patient's UNOS listing status (Table 1) is 2a, if it is 24–29, it is 2b, and if it is less than 24, it is 3. 

The PELD score includes parameters like albumin, bilirubin, INR, age (<1 year, >1 year), and the presence of growth failure tostratify children with liver disease on the waiting list.

## 2. Timing of Referral

Patients with a MELD score of >10 and/CTP score of >7 are referred for transplantation [10]. Other criteria to take into consideration are those with decompensated chronic liver disease in the form of intractable ascites, spontaneous bacterial peritonitis, variceal bleeding, encephalopathy, jaundice as well as health-related quality of life issues such as severe itching and recurrent cholangitis. Conditions which are not included in the scoring system and influence allocation are hepatocellular carcinoma, hepatopulmonary syndrome, and portopulmonary hypertension (Tables 2 and 3). Organ allocation is according to the status of the patient (UNOS status) and the MELD/PELD score. A Status 1 (Table 1) patient is given priority following which those with a MELD/PELD score ≥15 and later those having a score of ≤14.

## 3. Indications for Liver Transplantation

The list of indications for liver transplantation includes all the causes of end stage liver disease which are irreversible and curable by the procedure (Tables 2 and 3). In 1997 the American Society of Transplant Physicians and the American Association for the Study of the Liver Disease put forward the minimal listing criteria for patients with end stage liver disease. To qualify for the listing, the patient's expected survival should be ≤90% within 1 year without transplantation. Liver transplantation should lead to prolonged survival and an improved quality of life [10]. The outcome following liver transplantation is better for those with chronic cholestatic liver disease (including primary biliary cirrhosis and primary sclerosing cholangitis) compared with those who have hepatocellular carcinoma. 

### 3.1. Acute Liver Failure (ALF)

Fulminant hepatic failure (ALF and subfulminant hepatic failure) is characterized by encephalopathy, jaundice, and coagulopathy. It accounts for 5-6% of all patients undergoing liver transplantation [4]. In the West, acetaminophen toxicity is the leading cause of ALF, and hepatitis A, E, B and seronegative hepatitis are the other common aetiological factors. The major cause of subfulminant hepatic failure is idiosyncratic drug induced liver injury [11]. Patients who meet the King's College Criteria for urgent transplantation provide a very small window for action, and they need to undergo transplantation, as soon as possible. There is a 100% percent mortality if these selected patients do not undergo transplantation and this is either due to liver failure *per se* or because of sepsis and multiorgan failure [11]. Patients with subacute failure have a poor outcome with almost universal mortality if not transplanted; these patients might require transjugular liver biopsy to establish the presence of massive or submassive liver cell necrosis. Timely referral is important in these patients because in the absence of transplantation death may occur from sepsis and cerebral oedema. There are several scoring systems for listing a patient for urgent liver transplantation: King's College criteria, UK Blood and Transplant criteria, Clichy criteria (acute viral hepatitis), and Wilson's prognostic index/revised Wilson's prognostic index (Wilson's disease with fulminant hepatitis) [12–16]. (Tables 4 and 5).

### 3.2. Chronic Liver Disease

Patients who have a projected 1-year mortality of 10% without liver transplantation get entry into the waiting list. Apart from their CTP and MELD scores, the UK Liver Transplant Units have developed a new scoring system to predict the mortality of such patients. This is the United Kingdom model for end-stage liver disease (UKELD) score—which is calculated by using the patient's serum bilirubin, INR, creatinine, and sodium levels [17]. Patients with a UKELD score of more than 49 fall into the criteria for listing. This score is dynamic and is reassessed over a period of time.

### 3.3. Alcoholic Liver Disease (ALD)

A patient with ALD who is abstinent for a period of at least 3–6 months and who has had an evaluation with a psychiatrist is listed for transplantation if he has a CTP score of ≥7, portal hypertensive bleed, or an episode of spontaneous bacterial peritonitis [18]. These patients may have a concurrent infection with hepatitis B or C virus which needs evaluation. They are also more prone to develop hepatocellular carcinoma. A period of abstinence is mandatory to ensure that they do not relapse and also to give a trial of an alcohol-free period during which the liver function might recover. The period of abstinence is not uniform, however, but presently a 6-month rule of abstinence is generally followed in US and European liver transplant programmes [19].

Acute alcoholic hepatitis (AAH) is a contra-indication for liver transplantation as the required period of abstinence is lacking, and there is very little and mixed experience of liver transplantation in this situation. The severity of AAH is assessed using the Maddrey discriminant function (DF) score which predicts the risk of early death. Patients with a DF score of ≥32 are put on medical therapy [20, 21]. There have been recent reports from France where transplantation is being proposed for patients with AAH; however, it is still not accepted as an indication elsewhere [22].

### 3.4. Viral Hepatitis

Hepatitis C virus (HCV)-related chronic liver disease is the commonest indication for liver transplantation in the United States [23]. It is important to know the pretransplant viral load and genotype; this helps in predicting the prognosis after transplantation. Patients with decompensated HCV-related chronic liver disease do not tolerate interferon therapy, and those with high viral loads have a high chance of recurrence in the new graft. According to the International Liver Transplantation Society (ILTS) guidelines patients with a child's score of 8–11 may be considered for antiviral treatment while they are listed for transplantation; however, there are very high chances of adverse events [24]. Posttransplantation serological recurrence is universal in patients who have viraemia at the time of transplantation. Patient survival is adversely affected by the pretransplant viral load and cytomegalovirus status, advanced recipient age, hyperbilirubinaemia, a raised INR, and advanced donor age [25]. Retransplantation in these patients with recurrent HCV infection and cirrhosis is controversial in the setting of DDLT. The efficacy of antiviral therapy in the presence of a recurrence is questionable. Patients with early (within one year) aggressive recurrence and graft failure have a poor outcome following retransplantation.

Hepatitis B virus-related chronic liver disease is another common indication for transplantation, and this was previously also associated with a high prevalence of recurrent infection in the graft. However, the availability of hepatitis B immunoglobulin (HBIG) and oral nucleoside or nucleotide therapy reinfection of the graft and recurrent hepatitis B disease is rare. The duration of HBIG therapy and oral antiviral therapy is still controversial; a few programmes give HBIG for one year while others are using it life long [26].

### 3.5. Cholestatic Liver Disease

The severity of cholestatic liver diseases such as primary biliary cirrhosis(PBC) and primary sclerosing cholangitis (PSC) is taken into consideration apart from using the child's score (≥7) and the Mayo models for PSC and PBC with a risk score predicting > than 10% mortality at one year without transplantation [10]. Quality of life issues like recurrent cholangitis requiring repeated drainage procedures (endoscopic or percutaneous), intractable itching, xanthomatous neuropathy, and severe metabolic bone disease are some of the other indications for transplantation.

In paediatric patients, biliary atresia and sclerosing cholangitis are the commonest cholestatic disorders requiring transplantation, with biliary atresia being the foremost cause (60–70%) in those undergoing liver transplantation [27]. Liver transplantation is required in most patients with biliary atresia irrespective of a previous Kasai's procedure. Other cholestatic disorders which can lead to cirrhosis and decompensation requiring transplantation are the Alagille syndrome and Byler's disease.

### 3.6. Hepatic Malignancy

Cirrhosis is associated with a 2 to 8% annual incidence of hepatocellular carcinoma [28]. Liver transplantation has become the mainstay of treatment for HCC in the early stages, as it offers the advantage of not only being curative, thus, minimizing the risk of recurrence; it also takes care of the complications associated with the underlying cirrhosis. There have been several criteria for listing these patients for transplantation. They have been modified over a period of time so as to include as many patients who would benefit from transplantation and who would have a 5-year survival of >50%. The Milan criteria defines early stage HCC as those with a single lesion < 5 cm, or no more than 3 lesions, with none > than 3 cm, in the absence of vascular invasion and metastases [29]. However, using the University of California, San Francisco, (UCSF) criteria (a single lesion ≤6.5 cm or 3 or fewer lesions with the largest being ≤4.5 cm and a total tumour burden of 8 cm or less), patients had a similar outcome following transplantation compared to those within the Milan criteria [30]. The MELD score in patients with HCC might be low, and this might prevent these patients from being given priority or even being listed in spite of the fact that their disease is fatal if left untreated. Because this these patients are prioritized depending upon the stage of the tumour, those with T1 lesions are given a score of 20, and T2 lesions a score of 24 [31]. While waiting for transplantation, they usually undergo either transarterial chemoembolisation or radiofrequency ablation as a “bridge” to more definitive therapy.

Other uncommon primary malignancies of the liver which are indications for transplantation are epitheloid haemangioendothelioma and hepatoblastoma. Metastatic lesions of the liver have a poor prognosis; hence, they do not form an indication for transplantation; however, neuroendocrine tumors after the removal of the primary may have a good outcome following the procedure.

### 3.7. Metabolic Liver Disease

Metabolic liver diseases which cause decompensation and irreversible damage are indications for transplantation. These include Wilson's disease, hereditary haemochromatosis, and *α*_1_-antitrypsin disease. They also affect other organ systems; hence, pretransplant evaluation includes assessment of the concerned system to rule out systemic disease which would otherwise preclude transplantation. Other metabolic disorders, which affect extrahepatic organs while the synthetic liver functions are intact like Type-1 hyperoxaluria or familial homozygous hypercholesterolaemia, are indications for transplantation as the concerned metabolic disorder gets corrected. In childhood, the metabolic disorders which form an indication for transplantation are the urea cycle defects, Criggler-Najjar syndrome, tyrosinaemia, and cystic fibrosis.

### 3.8. Vascular Disorders

The Budd-Chiari syndrome is characterized by obstruction to the hepatic venous outflow either at the level of the hepatic veins and/or the inferior vena cava. It is associated with myeloproliferative disorders (50%), malignancy (10%), hypercoagulable states (15%), webs in the IVC, and paroxysmal nocturnal haemoglobinuria (5%). No cause is found in about 20% of patients. Indications for transplantation in these patients are established cirrhosis and acute decompensation. These patients generally require life-long anticoagulation after the transplant procedure [32].

### 3.9. Miscellaneous

Complicated polycystic liver disease (combined with or without kidney disease) with haemorrhage, infection, pain, massive cystic enlargement, portal hypertension, biliary obstruction, and rarely malignant transformation also forms an indication for liver transplantation. These patients might have well-preserved synthetic functions. Auto immune hepatitis (AIH) either alone or as an overlap syndrome with PSC/PBC is another indication for transplantation. It is important to identify the AIH as these patients require life-long low-dose steroids. Nonalcoholic steatohepatitis is another cause of cirrhosis which might require transplantation.

## 4. Contraindications to Liver Transplantation

### 4.1. Severe Cardiopulmonary Disease

Severe pulmonary hypertension or hypoxaemia resulting from the hepatopulmonary syndrome (HPS) poses an undue risk to patients at the time of transplantation (Table 6). A mean pulmonary arterial pressure (PAP) of ≥50 mmHg is an absolute contra-indication for transplantation as the postprocedure mortality is 100%. Those with PAP between 35–50 mmHg have a 50% mortality after transplantation. Patients with mild pulmonary hypertension with a mean PAP of <35 mmHg are suitable candidates [33]. The mortality in patients with HPS increases to about 30% in the presence of arterial hypoxaemia (<50 mmHg PaO_2_) [34]. Oxygen-dependent chronic obstructive airways disease and advanced pulmonary fibrosis are contraindications for transplantation, whereas reactive airway disease, hepatic hydrothorax, muscle wasting and infection, being reversible conditions, are only relative contraindications.

Symptomatic coronary artery disease, severe ventricular dysfunction, advanced cardiomyopathy, severe valvular heart disease, and aortic stenosis having poor ventricular function are absolute contraindications for transplantation. Following bypass surgery or revascularization and angioplasty wherein myocardial ischaemia is resolved, these patients could be listed for transplantation.

### 4.2. Active Alcohol and Substance Abuse

Active alcohol intake or substance abuse is absolute contraindication for transplantation. A pretransplant period of abstinence is a must for listing in most transplant programmes, but the period of abstinence is not well defined (6 months is generally required) [35]. This period of abstinence gives time for the acute insult on the liver to recover (if at all some recovery takes place); it also provides an opportunity for psychosocial assessment and preparation to minimize the chance of recidivism following transplantation. About 20–26% of patients resume heavy alcohol intake within 4.5 years of transplantation; this affects the graft survival adversely [36].

Acute alcoholic hepatitis is a contraindication for transplantation in almost all programmes. There is insufficient data on the outcome of transplantation in these patients as there is no period of abstinence [20, 21]. 

It is essential to rule out drug or poly drug abuse (opiates, sedatives, and cannabinoids), active tobacco abuse, as they form a high-risk group requiring psychiatric assessment and treatment. These candidates have a relative contraindication to be listed for transplantation as long as the active abuse continues.

### 4.3. Psychosocial Support

Patients following transplantation require good social support, in the absence of which it is likely that there will be lack of compliance with the immunosuppressive medication leading ultimately to the loss of the graft.

### 4.4. Age

Advanced age is associated with cardiopulmonary risk factors. Older patients require extensive evaluation to rule out the absolute contraindications like severe cardiopulmonary disease and malignancy. Patients over the age of 60–65 have been shown to have lower survival rates at 1 year and 5 years than those who are younger [37]. However, many centres now accept 70 years as the cut off limit for transplantation and have shown good results with this policy [38].

### 4.5. Obesity

Morbidly obese patients (BMI > 40) have an increased 5-year mortality after transplantation because of the associated cardiovascular morbidity [39]. Recipients who have a BMI > 35 kg/m^2^ require an individualized approach according to the policy of the centre.

### 4.6. HIV Infection

Patients with HIV infection have a better survival due to the effectiveness of highly active antiretroviral therapy (HAART). However they have other complications which may prove fatal, like chronic hepatitis C and cirrhosis. Earlier HIV used to be an absolute contraindication for transplantation, due to the fear of progression of disease with immunosuppression; however, with the availability of highly effective antiretroviral drugs, virus control has improved and transplantation is now being offered selectively. The absolute contraindication to transplantation in these patients includes uncontrolled HIV disease with multi drugresistance, leukoencephalopathy, advanced malnutrition, life support requirement, and opportunistic infections [26]. Transplantation in these patients should be done in collaboration with experts in the management of HIV infections.

### 4.7. Other Infections

Pneumonia, sepsis, bacteraemia, osteomyelitis, and fungal infection should be treated adequately before transplantation and the ongoing presence of any of these is an absolute contra-indication.

### 4.8. Miscellaneous

Retransplantation for recurrent HCV infection, autoimmune hepatitis, alcoholic liver disease, nonalcoholic steatohepatitis, and hepatocellular carcinoma are some of the controversial areas though not contraindications in themselves. This is because the survival of both patient and graft is suboptimal in the long term. Previous abdominal surgery increases the length of operation, blood loss, and complications related to the transplantation procedure. Portal vein thrombosis, once considered to be a contraindication, is no longer so except in the presence of diffuse thrombosis [40]. Patients with extrahepatic malignancy require at least a 5-year tumour-free interval before being considered for transplantation [41]. Cholangiocarcinoma once an indication for liver transplantation is now a relative contraindication because of the poor outcome especially in those with advanced disease.

## 5. Delisting Criteria

While waiting for the graft, if the liver disease progresses to such an extent that the survival benefit from transplantation (50% 5 year survival) no longer holds, which generally occurs if the MELD score is >40, then it is probably better to delist the patient. However, there is no guideline as such for delisting candidates except in patients with HCC who develop metastatic disease and fall out of the listing criteria. Patients who resume alcohol intake or substance abuse should be delisted. Temporary deactivation is done for patients who have clinical deterioration in the form of mechanical ventilation, haemodialysis, and fungal or resistant bacterial infection.

## 6. Living Donor Liver Transplantation

The indications for liver transplantation and the criteria for listing generally remain the same (child's score ≥7, MELD >10). Patients with cholestatic liver disease who have lower MELD scores, but other issues like, recurrent cholangitis, recurrent encephalopathy, and severe itching, who might not get listed in a DDLT program may, however, gain entry into the list in an LDLT setting. These patients benefit from partial liver grafts as they have otherwise stable liver disease. Studies have revealed that the average MELD score in a patient having LDLT is less than the score of a DDLT recipient (14.8 versus 23.5) [8]. The risk of transplantation is increased compared with its benefit if the MELD score is <14 or more than 25 [8]. There are two situations where LDLT poses an added advantage over DDLT. The first is a patient with HCC who probably has a lower MELD score, the waiting period is reduced, and the outcome is equally good. Patients fulfilling the Milan or UCSF criteria, depending upon the programme, get transplanted earlier in an LDLT setting before metastases occur. The other patients who have low MELD scores and would benefit from LDLT are those with symptomatic benign liver lesions (haemangioma, haemagioendothelioma, and polycystic liver disease), metabolic disorders (familial amyloidosis, hyperoxaluria, tyrosinaemia, glycogen storage disease), or complicated cholestatic liver disease. These patients otherwise would have to wait for a longer period to get a deceased donor graft.

The advantages of LDLT are that almost all transplants are planned and elective (except for those with ALF), the recipient's functional status can be optimized before surgery, and the graft cold ischaemia time is reduced. There has been evolution in the donor and patient selection, along with improvement in the surgical technique in both the donor and recipient surgery in LDLT. This has led to improved 1-year graft and patient survival to 81 and 89%; the vascular and biliary complications have reduced (hepatic artery thrombosis <5%, biliary complications 5–20%) [42–45]. 

It is very important to ensure donor safety in an LDLT program, and so far the reported donor mortality is <0.2–0.5%, morbidity is between 10–15%, and donor biliary complication is <5% [42, 46].

## 7. Contraindications for LDLT

Apart from the contraindications already mentioned in the earlier section, the additional contraindications pertaining to the living donor are as stated below.

**Absolute Contraindications**

1. Donor having macrosteatosis (>20%) on liver biopsy are rejected.
2. Remnant liver volume less than 25%. This is an issue especially when right lobe graft is big. It is never an issue when the left lateral segment is the proposed graft and is rarely an issue if the left lobe graft is taken.
3. The Human Organ Transplantation Act, in India, does not allow unrelated donation; this is to prevent donation under any kind of coercion and to avoid any organ trade. However unrelated donation is acceptable in other countries like Hong Kong, Korea, China, Japan, and so forth.
4. Living donor should be between 18 and 55 years of age. Lower limit is the age at which legal consent can be given.

**Relative Contraindications**

1. Body mass index >30 of the donor is generally associated with macrosteatosis, such donors are advised to reduce weight, and they need to have a liver biopsy to rule out >20% steatosis. If there are other potential donors in the family, they are rejected as liver donors.
2. Liver attenuation index of <5 on plain CT scan is suggestive of steatosis; hence, such donors are either rejected or in the absence of other donors need to reduce weight and have a biopsy to rule out >20% macrosteatosis.
3. Donors are rarely rejected on anatomical grounds. Double artery, double portal vein, or more than 2 hepatic veins can be easily tackled during implantation, and these no longer preclude donation. However, certain anatomical anomalies, for example, a Type E portal vein in the donor where there are multiple right-sided segmental portal vein tributaries draining into the left portal vein is a contraindication for LDLT [47].
4. All types of biliary anatomy in the donor (as classified by Huang) is acceptable [48]. Very rarely if there are multiple ducts in the donor (more than 3 bile ducts) to be anastomosed, then the donor is rejected [45].

## 8. Summary

Survival after liver transplantation has progressively improved over the decades. This can be attributed to advances in the surgical technique, perioperative and post-transplant intensive care management, and the introduction of better immunosuppressive drugs. Thus, there has been a constant evolution in the indications and contraindications for liver transplantation. Better scoring systems have been introduced to select patients and allocate organs in DDLT programs. Living donor transplantation has been introduced to overcome the gap between the need and availability of deceased donor organs especially in countries where deceased organ donation is rare. It offers definite advantage in situations where the waiting period otherwise would be lengthy and where entry to the waiting list for a DDLT would be restricted.

## Figures and Tables

**Table 1:** United network for organ-sharing (UNOS) liver status classification.

Status 1	Fulminant liver failure with life expectancy <7 days
	(i) Fulminant hepatic failure as traditionally defined
	(ii) Primary graft nonfunction <7 days of transplantation
	(iii) Hepatic artery thrombosis <7 days of transplantation
	(iv) Acute decompensated Wilson's disease

Status 2a	Hospitalized in ICU for chronic liver failure with life expectancy <7 days, with a Child-Pugh score of ≥10 and one of the following:
	(i) unresponsive active variceal hemorrhage
	(ii) hepatorenal syndrome
	(iii) refractory ascites/hepatic hydrothorax,
	(iv) Stage 3 or 4 hepatic encephalopathy

Status 2B	Requiring continuous medical care, with a Child-Pugh score of ≥10, or a Child-Pugh score ≥7 and one of the following:
	(i) unresponsive active variceal hemorrhage
	(ii) hepatorenal syndrome
	(iii) spontaneous bacterial peritonitis
	(iv) refractory ascites/hepatic hydrothorax,
	or presence of hepatocellular carcinoma

Status 3	Requiring continuous medical care, with a Child-Pugh score of ≥7, but not meeting criteria for Status 2B

Status 7	Temporary inactive

From http://www.unos.org/ initially implemented in July 1997 later modified in January 1998 and August 1998.

**Table 2:** Indications for liver transplantation.

*Acute liver failure*
Hepatitis A, acetaminophen, autoimmune hepatitis
Hepatitis B
Hepatitis C, cryptogenic
Drugs, hepatitis D
Wilson's disease, Budd-Chiari syndrome
Fatty infiltration—acute fatty liver of pregnancy, Reye's syndrome

*Cirrhosis from chronic liver disease*
Chronic hepatitis B virus infection
Chronic hepatitis C virus infection
Alcoholic liver disease
Autoimmune hepatitis
Cryptogenic liver disease
Nonalcoholic fatty liver disease

*Malignant diseases of the liver*
Hepatocellular carcinoma
Carcinoid tumor
Islet cell tumor
Epithelioid hemangioendothelioma
Cholangiocarcinoma

*Metabolic liver disease*
Wilson's disease
Hereditary hemochromatosis
Alpha-1 antitrypsin deficiency
Glycogen storage disease
Cystic fibrosis
Glycogen storage disease I and IV
Crigler-Najjar syndrome
Galactosemia
Type 1 hyperoxaluria
Familial homozygous hypercholesterolemia
Hemophilia A and B

*Vascular diseases of the liver*
Budd-Chiari syndrome
Veno-occlusive disease

*Cholestatic liver diseases*
Primary biliary cirrhosis
Primary sclerosing cholangitis
Secondary biliary cirrhosis
Biliary atresia
Alagille syndrome
Byler's disease

*Miscellaneous*
Adult polycystic liver disease
Nodular regenerative hyperplasia
Caroli's disease
Severe graft-versus-host disease
Amyloidosis
Sarcoidosis
Hepatic trauma

**Table 3:** Variant syndromes requiring liver transplantation.

*Intractable ascites*
Diuretic resistant, Nonresponsive to TIPS or, TIPS
contraindicated
*Hepatopulmonary Syndrome*
Shunt fraction >8%, pulmonary vascular dilatation
*Chronic hepatic encephalopathy*
*Persistent and intractable pruritus*

**Table 4:** UK blood and transplant criteria for registration as a super-urgent transplant.

Paracetamol poisoning	
Category 1	pH < 7.25 more than 24 hours after overdose and fluid resuscitation

Category 2	Coexisting prothrombin time >100 s or INR > 6.5 and serum creatinine >300 *μ*mol/L or anuria, and grade 3-4 en encephalopathy

Category 3	Serum Lactate >24 hours after overdose > 3.5 mmol/L on admission or >3 mmol/L after fluid resuscitation

Category 4	Two of the three criteria from category 2 with clinical evidence of deterioration (e.g. increased ICP, Fi02 > 50%, increasing inotrope requirement) in the absence of clinical sepsis

Seronegative hepatitis, hepatitis A, B, or an idiosyncratic drug	
reaction	

Category 5	Prothrombin time >100 s or INR > 6.5, and any grade of encephalopathy

Category 6	Any grade of encephalopathy, and any three from the following: unfavourable aetiology (idiosyncratic drug reaction, seronegative hepatitis), age > 40 years jaundice encephalopathy interval >7 days, serum bilirubin >300 *μ*mol/L, prothrombin time >50 s or INR > 3.5

Category 7	Acute presentation of Wilson's disease, or Budd-Chiari syndrome. A combination of coagulopathy, and any grade of encephalopathy

Category 8	Hepatic artery thrombosis on days 0 to 21 days after liver transplantation

Category 9	Early graft dysfunction on days 0 to 7 after liver transplantation with at least 2 of the following: AST > 10,000 IU/L, INR > 3.0, serum lactate > 3 mmol/L, absence of bile production

Category 10	Any patient who has been a live donor who develops severe liver failure within 4 weeks of the donor operation

**Table:** (a) King's College Criteria

**Acetaminophen-induced ALF**	**Nonacetaminophen ALF**
(1) Arterial pH < 7.3 *irrespective* of grade of encephalopathy	(1) INR > 6.5 (PT > 100 sec), *irrespective* of grade of encephalopathy
OR	OR any 3 of the following:
(1) PT > 100 sec	(1) INR > 3.5 (PT > 50 sec)
(2) Serum creatinine >3.4 mg/dL	(2) Age < 10 or >40 years
(3) Stage 3 or 4 encephalopathy	(3) Serum bilirubin >18 mg/dL
	(4) Jaundice to encephalopathy interval >7 days
	(5) Non-A, non-B hepatitis, idiosyncratic drug reaction

**Table:** (b) Prognostic index in fulminant Wilsons hepatitis (WPI) [14]

Score	0	1	2	3	4
Serum bilirubin (reference range 3–20 mmol/L)	<100	100–150	151–200	201–300	>300
Serum aspartate transaminase (reference range 7–40 IU/L)	<100	100–150	151–200	201–300	>300
Prothrombin time prolongation (seconds)	<4	4–8	9–12	13–20	>30

*Patients with a WPI score ≥7 need urgent liver transplantation*					

**Table:** (c) Revised Wilson prognostic index (RWPI) [15]

Score	Bilirubin (*μ*mol/L)	INR	AST (IU/L)	WCC (10^9^/L)	Albumin (g/L)
0	0–100	0–1.29	0–100	0–6.7	>45
1	101–150	1.3–1.6	101–150	6.8–8.3	34–44
2	151–200	1.7–1.9	151–300	8.4–10.3	25–33
3	201–300	2.0–2.4	301–400	10.4–15.3	21–24
4	≥301	>2.5	>401	>15.4	<20

*Patients with a RWPI ≥11 needed urgent liver transplantation*					

**Table:** (d) Clichy criteria (Hospital Paul-Brousse, Villejuif [16])

Hepatic encephalopathy, and factor V level:
<20% in patients <30 years of age, or
<30% in patients ≥30 years of age

**Table 6:** Contraindications to liver transplantation.

*Absolute contraindications*
Severe cardiopulmonary disease
Extrahepatic malignancy (oncologic criteria for cure not met)
Active alcohol/substance abuse
Acute alcoholic hepatitis
Active infection/uncontrolled sepsis
Lack of psychosocial support/inability to comply with medical
treatment
Brain death
*Relative contraindications*
Advanced age
Acquired immune deficiency syndrome
Cholangiocarcinoma
Diffuse portal vein thrombosis
